# Effect of (R)‐salbutamol on the switch of phenotype and metabolic pattern in LPS‐induced macrophage cells

**DOI:** 10.1111/jcmm.14780

**Published:** 2019-11-03

**Authors:** Shanping Wang, Fei Liu, Keai Sinn Tan, Hooi‐Leng Ser, Loh Teng‐Hern Tan, Learn‐Han Lee, Wen Tan

**Affiliations:** ^1^ School of Biology and Biological Engineering South China University of Technology Guangzhou China; ^2^ Institute of Biomedical and Pharmaceutical Sciences Guangdong University of Technology Guangzhou China; ^3^ Novel Bacteria and Drug Discovery (NBDD) Research Group Microbiome and Bioresource Research Strength Jeffrey Cheah School of Medicine and Health Sciences Monash University Malaysia Bandar Sunway Malaysia; ^4^ Jeffrey Cheah School of Medicine and Health Sciences Monash University Malaysia Bandar Sunway Malaysia

**Keywords:** cell metabolomics, ECAR, inflammation, macrophage polarization, OCR

## Abstract

Evidence demonstrates that M1 macrophage polarization promotes inflammatory disease. Here, we discovered that (R)‐salbutamol, a β_2_ receptor agonist, inhibits and reprograms the cellular metabolism of RAW264.7 macrophages. (R)‐salbutamol significantly inhibited LPS‐induced M1 macrophage polarization and downregulated expressions of typical M1 macrophage cytokines, including monocyte chemotactic protein‐1 (MCP‐1), interleukin‐1β (IL‐1β) and tumour necrosis factor α (TNF‐α). Also, (R)‐salbutamol significantly decreased the production of inducible nitric oxide synthase (iNOS), nitric oxide (NO) and reactive oxygen species (ROS), while increasing the reduced glutathione (GSH)/oxidized glutathione (GSSG) ratio. In contrast, (S)‐salbutamol increased the production of NO and ROS. Bioenergetic profiles showed that (R)‐salbutamol significantly reduced aerobic glycolysis and enhanced mitochondrial respiration. Untargeted metabolomics analysis demonstrated that (R)‐salbutamol modulated metabolic pathways, of which three metabolic pathways, namely, (a) phenylalanine metabolism, (b) the pentose phosphate pathway and (c) glycerophospholipid metabolism were the most noticeably impacted pathways. The effects of (R)‐salbutamol on M1 polarization were inhibited by a specific β_2_ receptor antagonist, ICI‐118551. These findings demonstrated that (R)‐salbutamol inhibits the M1 phenotype by downregulating aerobic glycolysis and glycerophospholipid metabolism, which may propose (R)‐salbutamol as the major pharmacologically active component of racemic salbutamol for the treatment of inflammatory diseases and highlight the medicinal value of (R)‐salbutamol.

## 1 INTRODUCTION

Inflammation occurs when immune receptors recognize damaged cells and pathogens. Under normal physiological conditions, inflammation is a protective response to internal injury or external pathogens.^1^ However, an excessive inflammatory response is associated with the pathogenesis of many human diseases.^2^, ^3^ As the most abundant innate immune cells, macrophages are critical agents in inflammatory disease. Depending on the type of stimuli, macrophages show phenotypic heterogeneity and have different functional activities. Recent studies have shown that the relative proportion of macrophage subsets, rather than the absolute number of macrophages, significantly affects disease progression.^4^, ^5^

By combining gene expression profiles and surface marker quantification, a series of different macrophage populations, namely M1 pro‐inflammatory and M2 anti‐inflammatory macrophages, have been characterized. M1 macrophages mediate inflammation by upregulating monocyte chemotactic protein (MCP)‐1, interleukin (IL)‐1β and tumour necrosis factor‐alpha (TNF‐α), while M2 macrophages mediate the resolution of inflammation by inducing the expression of CD206, arginase‐1 (arg‐1) and IL‐10.^6^ Failure to regulate these mediators can lead to tissue damage and cell destruction due to production of reactive oxygen species (ROS) and nitric oxide (NO). Pro‐inflammatory cytokines associated with M1 macrophages are increasingly recognized as central mediators in chronic inflammatory diseases and some cardiovascular diseases (CVDs).^7^, ^8^, ^9^, ^10^ The persistent polarization of M1 macrophages leads to an inflammatory milieu that prevents the transition to inflammation regression.^11^ It is well known that redox imbalance and oxidative stress contribute to the inflammatory development. Glutathione (GSH), low‐molecular weight thiol compound, can protect cells from oxidative stress by scavenging excess radicals.^12^ Therefore, inhibiting the polarization of M1 macrophages is key to reducing the level of inflammation in the progression of various diseases.

Metabolic changes in macrophages are associated with different inflammatory responses. Pathogen infection, for example can lead to the “reprogramming” of immune cell metabolism. There is an increase in intracellular glucose metabolism (increased aerobic glycolysis (ie Warburg effect) and accelerated glucose uptake) upon pathogen infection, which mobilizes immune cells to destroy foreign bodies.^13^ Other studies have reported that modifications of aerobic glycolysis result in altered immune cell activities.^14^ Taken together, these data suggest that immune cell activity may be moderated via intracellular glucose metabolism regulation.

β_2_ adrenergic receptor agonists are known treatments of obstructive lung diseases.^15^ The activation of adenylate cyclase increases cyclic AMP synthesis and relaxes bronchial smooth muscle. Moreover, β_2_ adrenergic receptor agonists possess a number of anti‐inflammatory effects.^16^ Racemic salbutamol is a 50:50 mixture of the (S)‐ and (R)‐isomers of salbutamol, of which the latter acts as the active enantiomer.^17^ (S)‐salbutamol was found to induce exaggerated airway reactivity and exacerbate asthmatic conditions.^18^, ^19^ It increases intracellular calcium, causing airway hypersensitivity and leading to bronchoconstriction,^18^ and thus may contribute to the cumulative adverse effects. In addition, salbutamol has been shown to modulate the macrophage immune response.^16^ (R)‐salbutamol exhibits anti‐inflammatory functions by modifying transcription factors and suppressing cytokine molecular cascades involved in inflammation, as well as ridding the cells of superoxide and peroxidase. However, the anti‐inflammatory mechanisms of (R)‐salbutamol are not fully understood, and the association of the anti‐inflammatory potential of (R)‐salbutamol with macrophage metabolism and polarization has yet to be investigated. The RAW264.7 cell line is an established model for the study of macrophage function and was used to investigate the in vitro effects of (R)‐salbutamol on macrophage polarization and metabolism.

In this study, we evaluated the effect of (R)‐salbutamol on the inhibition of the lipopolysaccharide (LPS)‐induced activation of RAW264.7 macrophages. We examined the effect of (R)‐salbutamol on the typical cytokines of M1 macrophages at the messenger RNA (mRNA) and protein levels. In addition, we investigated the suppressive effects of (R)‐salbutamol on M1 macrophage polarization and cellular metabolism reprogramming. These findings may provide evidence for (R)‐salbutamol to be a candidate drug in treating inflammatory diseases.

## 2 MATERIALS AND METHODS

### 2.1 Reagents

(R)‐salbutamol (>99% purity, 99.85% ee) and (S)‐salbutamol (>99% purity, 92.73% ee) were provided by Dongguan Key‐Pharma Biomedical Co., Ltd. LPS (*Escherichia coli* O111:B4), ICI‐ 118551 hydrochloride, fluorescent probes 3‐Amino,4‐aminomethyl‐2′,7′‐difluorescein diacetate (DAF‐FM DA) and 2′,7′‐dichlorodihydrofluorescein diacetate (DCFH‐DA) were bought from Sigma Chemical Co. The kits for cDNA synthesis, BCA protein assay, cell culture reagents and SYBR Green Supermix were from by Life Technologies Inc (Gibco). Methanol and acetonitrile were acquired from Fisher Chemical. Phycoerythrin (PE)‐conjugated anti‐mouse F4/80 (123110), fluorescein isothiocyanate (FITC)‐conjugated anti‐mouse CD206 (141704) and allophycocyanin (APC)‐conjugated anti‐mouse CD11c (117310) were procured from BioLegend. β‐actin antibody (# BF01980) was obtained from Affinity Biosciences. Inducible nitric oxide synthase (iNOS) mouse antibody (2982S) was obtained from Cell Signaling Technology. The enzyme immunoassay kits for MCP‐1, IL‐1β and TNF‐α were manufactured by Neobioscience. Beyotime Institute of Biotechnology supplied the Cell Counting Kit‐8 (CCK‐8). Rotenone/antimycin A, carbonyl cyanide 4‐(trifluoromethoxy) phenylhydrazone (FCCP) and oligomycin came from Seahorse Bioscience (Agilent Technologies, Inc).

### 2.2 Cell culture and M1 macrophage polarization

RAW264.7 cell lines were gifted from the Southern Medical University. DMEM supplemented with 0.1% (v/v) penicillin/streptomycin, 10% (v/v) heat‐inactivated FBS and 4.5 g/L glucose was used to nurture the cells in a humidified incubator (5% (v/v) CO_2_ at 37°C), and cells at passages 5‐10 were used for all experiments. It was reported that treatment with 100 ng/mL LPS for 12 hours was found enough to induce the largest mRNA expressions of IL‐12, IL‐1, TNF‐α, IL‐1Ra, IL‐6 and IFN‐γ,^20^ This is consistent with another study indicating that treatment with 100 ng/mL LPS for 12 hours upregulated M1 macrophage cytokines.^21^ Based on these studies, a concentration of 100 ng/mL LPS and 12‐hour treatment period were selected to induce M1 polarization in RAW264.7 cells for subsequent experiments.

### 2.3 Cell viability assay

A CCK‐8 assay (Dojindo) was used to determine the cell viability following the manufacturer’s instructions. RAW264.7 cells were treated with various concentrations of (R)‐salbutamol for one hour prior to LPS induction (100 ng/mL). Cells were then incubated for a further 2 hours with the addition of 10 μL of CCK‐8. Cells were visualized at 450 nm with an Enspire‐2300 Multimode Reader (PerkinElmer).

### 2.4 Cell phenotype identification

Six‐well plates were used for seeding RAW264.7 cells (1 × 10^5^ cells/well) overnight. LPS (100 ng/mL) was used to treat the cells after the addition of (R)‐salbutamol. Upon completion of treatment, all cells were extracted and rinsed twice with PBS, before being blocked on ice for 30 minutes with magnetic‐activated cell sorting (MACS) buffer. Then, the cells were labelled with the following antibodies: PE‐conjugated anti‐mouse F4/80, APC‐conjugated anti‐mouse CD11c and FITC‐conjugated anti‐mouse CD206. FITC‐, APC‐ and PE‐conjugated rat anti‐mouse IgG antibodies served as an isotype control for nonspecific background signals. Labelled cells were analysed using a BD FACSAriaIII cell sorter (BDIS). FlowJo software (Tree Star, Inc) was used to analyse data.

### 2.5 ROS and NO detection

The intracellular ROS levels were examined using DCFH‐DA (Life Technologies‐Thermo Fisher Scientific) before visualization with a LSM710 Laser Scanning Confocal Microscope (Carl Zeiss) to quantify the fluorescence signals of the oxidized product (2′,7′‐dichlorofluorescein, DCF).

The Griess assay (Beyotime) was used to evaluate the amount of NO in the culture supernatant by measuring the concentration of nitrite (a stable NO breakdown product). An NO^−^ sensitive fluorescence probe DAF‐FM DA (Sigma) was used to detect intracellular NO.^22^ DAF‐FM DA (10 μmol/L) was used to label the cells at 37°C for 30 minutes before they were washed thrice with PBS. Fluorescence was detected using a LSM710 Laser Scanning Confocal Microscope (scale bars, 100 μm) (Carl Zeiss).

### 2.6 Intracellular GSH/GSSG ratio determination

The total levels of intracellular total GSH and oxidized glutathione (GSSG) in the cells were measured using a total GSH and GSSG assay kit (Beyotime), respectively.

### 2.7 Evaluation of cytokine levels by enzyme‐linked immunosorbent assay

Mouse enzyme‐linked immunosorbent assay (ELISA) kits were used to determine the concentrations of MCP‐1, IL‐1β and TNF‐α in the cell supernatants (Neobioscience).

### 2.8 RNA isolation and real‐time PCR analysis

TRIzol reagent (Life Technologies Inc, Gibco) was used to isolate total RNA from RAW264.7 cells prior to cDNA synthesis using the M‐MLV 1st Strand Kit from Invitrogen. Quantitative real‐time PCR was performed with the SYBR Green Mix (Life Technologies Inc, Gibco). The relative expression level of each mRNA (MCP‐1, IL‐1β and TNF‐α) was compared against the levels of the endogenous protein *β‐actin* with the 2^−∆∆Ct^ cycle threshold method. Table S1 lists all gene sequences related to this experiment.

### 2.9 Western blotting analysis

Western blotting was used to assess the relative expression levels of iNOS in RAW264.7 cells. Briefly, total protein was extracted, and protein concentrations were then determined with the BCA kit (Thermo Scientific). Proteins were denatured and then subjected to 8% (v/v) sodium dodecyl sulphate‐polyacrylamide gel electrophoresis and transferred onto polyvinylidene difluoride membranes (Bio‐Rad, CA, USA) before being exposed to 5% (w/v) skim milk for 1 hour. The membranes were then incubated with primary antibodies against iNOS (1:1000; Cell Signaling Technology) and β‐actin (1:1000; Affinity Biosciences) overnight at 4°C. The membranes were subsequently incubated with the appropriate secondary antibodies for 1 hour at room temperature. The membranes were rinsed with 0.1% (v/v) Tween‐20 in Tris‐buffered saline between each step. Finally, the signals were detected by Image Lab software (Bio‐Rad Laboratories) after incubation with an enhanced luminescence kit (Thermo Scientific).

### 2.10 Seahorse analysis

The extracellular acidification rate (ECAR) and oxygen consumption rate (OCR) of the RAW264.7 cells were measured in real‐time using a Seahorse XF96 extracellular flux analyser (Agilent). The cells were seeded in an XF96 cell culture plate 2 days before the experiment and cultivated in a humidified atmosphere (5% (v/v) CO_2_ at 37°C). The next day, 200 µL of XF calibrator was inserted into all XF cartridge wells before being subjected to an overnight incubation at 37°C in a humidified atmosphere without CO_2_. One hour prior to the experiment, cells were rinsed with PBS, and then, XF assay medium was added to each well and then incubation at 37°C for 1 hour in a humidified atmosphere without CO_2_. For OCR analysis, 1 mmol/L sodium pyruvate, 2 mmol/L l‐glutamine and 10 mmol/L glucose were added into the XF assay medium. After measuring basal respiration, rotenone/antimycin A (1 μmol/L), carbonyl cyanide m‐chlorophenyl hydrazone (0.5 μmol/L) and oligomycin (1 μmol/L) were injected into each sequence to evaluate respiratory chain coupling and the maximal and nonmitochondrial oxygen consumption. In the ECAR assay, 2 mmol/L l‐glutamine was added to the XF assay medium. Glycolytic flux (glycolytic reserve, glycolytic capacity and glycolytic reserve) was assessed by sequentially adding 10 mmol/L glucose, 1 μmol/L oligomycin and 50 mmol/L 2‐deoxyglucose. The OCR and ECAR values were automatically calculated by the Seahorse XF‐96 software.

### 2.11 Untargeted metabolic profiling

#### 2.11.1 Metabolite extraction of cells

After incubation, cells were harvested and disrupted using a tissue grinder (SCIENTZ‐48). A three‐solvent biphasic system with a methyl‐T‐butyl‐ether:methanol:water (MTBE solution, v/v/v) at a volume ratio of 6:3:1 was used to extract metabolites in this study.^23^, ^24^, ^25^ A total of 40 µL of cell lysate was added to 160 µL of MTBE solution, and the sample was vigorously vortexed at 4°C for 30 minutes, followed by centrifugation (3000× g, 4°C, 30 minutes). Two extract fractions were generated: (a) an organic hydrophobic layer composed of MTBE and methanol and (b) a hydrophilic layer composed of methanol and water. These two extract fractions were dried under vacuum and resuspended in 0.1% (v/v) formic acid in water (45 µL) prior to analysis. For quality assurance, 60 µL aliquots of each sample were pooled as a quality control (QC) sample to provide an accurate depiction of metabolite range.^26^, ^27^ The blank was injected during the initial run to condition the column. To ensure injection precision, six replicated analyses were performed with the same QC sample. Method repeatability was evaluated across six various QC samples to evaluate the precision of the developed method. The stability of the system was assessed by evaluating one QC sample per five experimental samples across the analytical run.

#### 2.11.2 UHPLC/ESI‐TIMS TOF‐MS/MS data acquisition and analysis

Samples were analysed with an ultra‐high performance liquid chromatography coupled with electrospray ionization quadrupole time‐of‐flight mass spectrometry (UHPLC/ESI‐TIMS TOF‐MS/MS) system in negative and positive ion mode, using a Dionex UltiMate 3000 RSLC system (Thermo Scientific/Dionex, Netherlands) with an Acquity UPLC BEH‐C18 column (2.1 mm × 50 mm, 1.7 µm), coupled to a trapped ion mobility spectrometer and time‐of‐flight (TOF) mass spectrometer (Bruker Daltonics Inc). Tandem mass spectrometry (MS/MS) data were acquired by an AutoMS/MS scan experiment with a data‐dependent acquisition (DDA) model, allowing the selection of the precursor ion as the most intense peak during liquid chromatography‐mass spectrometry (LC–MS) analyses. All samples were kept at 4°C, and 5 µL of each sample was used for analysis.

#### 2.11.3 Sample injection description

Two extract fractions from biphasic extractions were generated from each sample,^28^ an organic layer and an aqueous layer. The first injection of 5 µL of the organic layer was followed by a second injection of 5 µL of the aqueous phase onto the same column for the gradient described above. However, the gradient had not yet begun, and the method lasted only 1 minutes without increasing the concentration of the mobile phase of acetonitrile (B solvent), ensuring that the organic phase of hydrophobic lipids remained at the head of the column. Immediately afterwards (via the next line in the sequence table), 5 µL of the aqueous phase was injected into the same column, and the complete gradient was executed.

### 2.12 Statistical analysis

The UHPLC/ESI‐TIMS TOF‐MS/MS data were analysed using software (Waters) for peak alignment, selection and normalization to determine the peak intensities for retention time (RT) and m/z data pairs. The potential biomarkers responsible for the discrimination between these groups were identified based on variable importance in projection (VIP) values > 1.0, *P* values < .05 and max fold change > 2. The resultant data matrices were exported to the EZinfo 3.0 software for principal component analysis (PCA), partial least square discriminant analysis (PLS‐DA) and orthogonal partial least square discriminant analysis (OPLS‐DA). MS/MS analysis was used to assign metabolite peaks, or the data were interpreted with available biochemical databases, such as HMDB, ChemSpider, LipidMAPS and KEGG. The Venn plot analysis was carried out using OmicShare tools (http://www.omicshare.com/tools). Pathway analysis was performed using the KEGG pathway database and MetaboAnalyst 4.0 software.

Statistical analyses were carried out using GraphPad Prism 5 software (GraphPad Software Inc). Additional statistical analyses determined the means ± standard deviation (SDs) of three or more independent experiments. The groups were compared by Student's *t* test and one‐way analysis of variance (ANOVA), followed post hoc Tukey's test for multiple groups comparison. A value of *P* < .05 was considered statistically significant.

## 3 RESULTS

### 3.1 (R)‐salbutamol inhibits the polarization of M1 macrophages in LPS‐induced RAW264.7 cells via the β_2_ adrenergic receptor

(R)‐salbutamol is a well‐known asthma bronchodilator (Figure S1A). (R)‐salbutamol cytotoxicity on RAW2647 cells was examined by assessing cell viability through a CCK‐8 assay. Our data showed no changes in cell viability even when the concentration of (R)‐salbutamol reached 100 μmol/L with or without LPS (100 ng/mL) (Figure S1B‐C). The levels of NO and ROS after pretreatment with (R)‐salbutamol at various concentrations (0.25, 0.5, 1, 2, 5, 10 and 100 µmol/L) were examined in LPS‐induced RAW264.7 cells. The results showed that treatment with LPS led to a significant NO and ROS elevation in macrophage cells as evidenced by increased fluorescence intensity, while treatment with various concentrations of (R)‐salbutamol prior to LPS exposure significantly reduced the amount of intracellular NO and ROS induced by LPS in a dose‐dependent manner (data not shown). The average peak plasma concentration after a single 4 mg tablet of salbutamol is 30‐60 nmol/L.^29^, ^30^ The peak plasma concentrations of salbutamol post‐inhalation are 7.5‐23 nmol/L.^31^ The concentration of salbutamol in the lungs is inevitably higher than that in the blood. (R)‐salbutamol is a eutomer of salbutamol. Therefore in view of the effect of (R)‐salbutamol in various concentration on NO and ROS, the concentration of (R)‐salbutamol (10 µmol/L) used in this study is acceptable and mirrors dosages administered in other studies on human airway epithelial cells.^32^

**Figure 1:**
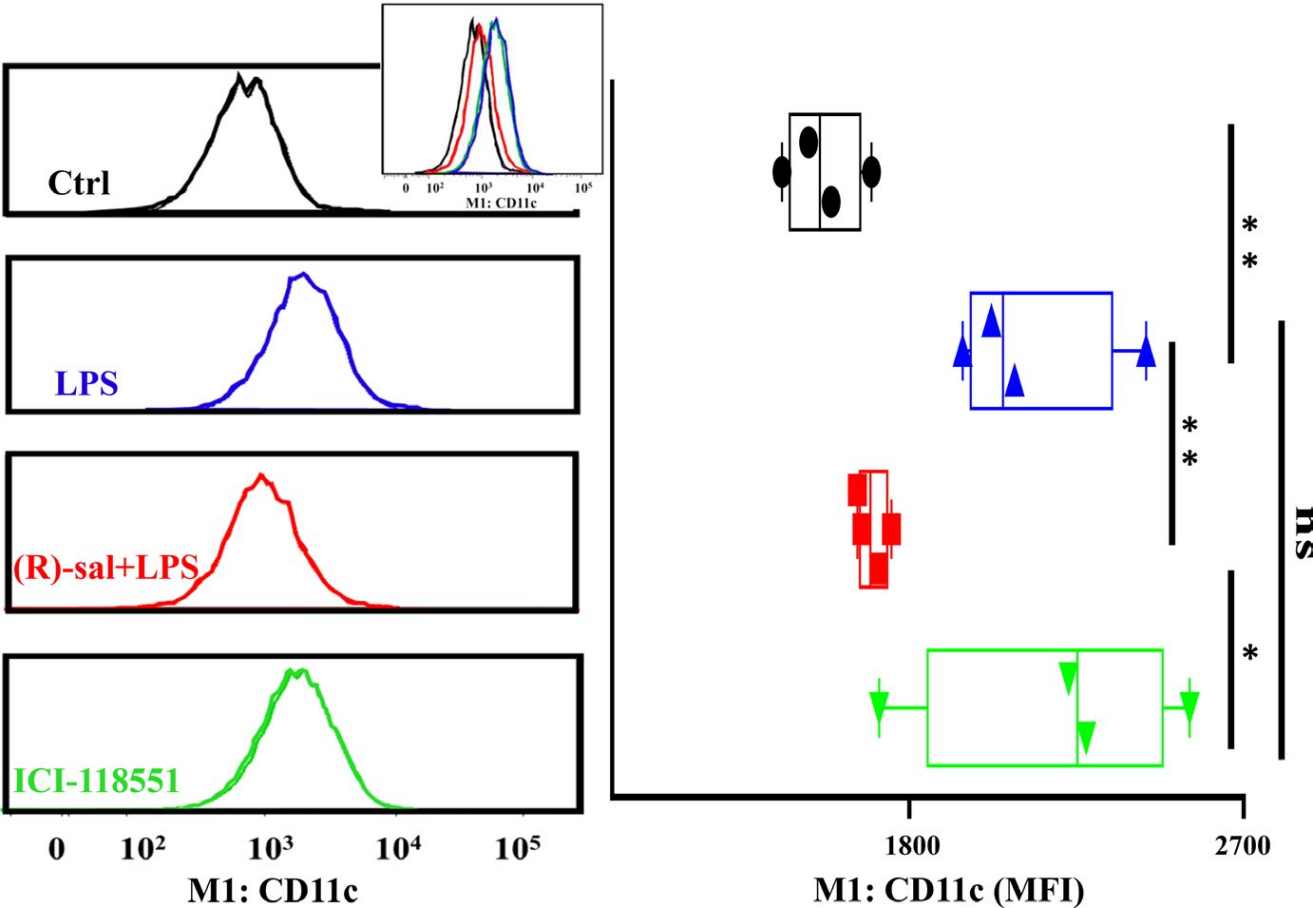
(R)‐salbutamol inhibits the polarization of M1 macrophages in LPS‐induced RAW264.7 cells. M1 macrophages expression histograms (left panels) and quantification (right panels) by flow cytometry in the control (Ctrl), LPS, (R)‐salbutamol and ICI‐118551 groups. Overlay histograms are shown (inset). The mean fluorescence intensity (MFI) decreased following (R)‐salbutamol treatment, but this effect was blocked by ICI‐118551. Data are expressed as the MFI. ^**^ *P* < .01, ^*^ *P* < .05; ns, not significant

Next, to investigate the effect of β_2_ adrenergic receptor activation on macrophage polarization, macrophages were pretreated with (R)‐salbutamol and subsequently induced with 100 ng/mL LPS. Flow cytometry was used to analyse the polarization of M1 and M2 macrophages. RAW264.7 macrophage surfaces contain the characteristic transmembrane protein F4/80, which is often used as an identification marker. F4/80‐positive cells were gated, and their M1 or M2 subtypes were further identified using CD11c and CD206 (Figure S2A). F4/80 + CD11c^+^CD206^−^ cells are defined as M1‐positive cells and F4/80 + CD11c^−^ CD206^+^ as M2‐positive cells.^33^ The cell distribution patterns of each group are shown in Figure S2B. Upon stimulation with LPS, the mean fluorescence intensity (MFI) and numbers of M1 macrophages increased to 73.9% and 97.7%, respectively, suggesting that LPS could induce M1 polarization. However, pretreatment with (R)‐salbutamol significantly reduced the MFI and counts of M1, indicating that (R)‐salbutamol alleviated the LPS‐induced polarization of M1 macrophage (Figures 1 and S2B). In the LPS‐induced macrophage cell model, only a few M2 macrophages can be observed. In addition, the MFI of M2 macrophages upon stimulation with LPS and pretreatment with (R)‐salbutamol were not significantly different. To investigate whether (R)‐salbutamol mediates its effects on M1 macrophage polarization via the β2 adrenergic receptor, a specific β2 adrenergic receptor antagonist, ICI‐118551, was employed in this study. These findings indicate that the MFI and counts of M1 macrophages were raised to 77.5% and 97.3%, respectively, when cells were pretreated with (R)‐salbutamol following incubation with ICI‐118551 (Figures 1 and S2B). Taken together, these findings suggested that (R)‐salbutamol exerted its inhibitory effects on M1 macrophage polarization through the β_2_ adrenergic receptor.

### 3.2 (R)‐salbutamol decreases the production of MCP‐1, IL‐1β and TNF‐α in LPS‐induced RAW264.7 cells

**Figure 2:**
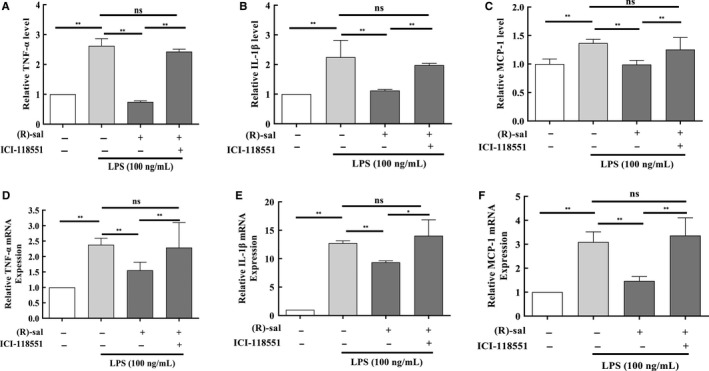
(R)‐salbutamol inhibits the expression of cytokines typically detected in M1 macrophages in LPS‐induced RAW264.7 cells. A‐B, The graph shows the protein expression levels of the pro‐inflammatory cytokines (A) TNF‐α, (B) IL‐1β and (C) MCP‐1 in the control (Ctrl), LPS, (R)‐salbutamol and ICI‐118551 groups as determined by ELISA. Ctrl cells served as a control. D‐F, Relative mRNA expression of the M1 macrophage markers (D) TNF‐α, (E) IL‐1β and (F) MCP‐1 were examined in the Ctrl, LPS, (R)‐salbutamol and ICI‐118551 groups by real‐time PCR. Data are presented as the mean ± SD. ^**^ *P* < .01, ^*^ *P* < .05; ns, not significant

To confirm that M1 polarization was more predominant than M2 polarization after stimulation with LPS, the levels of typical M1 macrophage cytokines (ie MCP‐1, IL‐1β and TNF‐α) were determined using ELISA; these cytokines are mainly synthesized by macrophages.^34^, ^35^ These pro‐inflammatory cytokines are mediators of many human chronic inflammatory diseases and have been associated with acute phase reactions.^34^, ^36^ As shown in Figure 2A‐C, 100 ng/mL LPS induced a significant increase in the amount of TNF‐α, IL‐1β and MCP‐1 in macrophages, whereas this effect was remarkably attenuated in (R)‐salbutamol treatment groups. We conclude that LPS is likely to lead to M1 polarization but not M2 polarization and (R)‐salbutamol inhibits M1 macrophages polarization.

Quantitative real‐time PCR was carried out to examine MCP‐1, IL‐1β and TNF‐α expression. Consistent with the cytokine expression study, the mRNA expression of TNF‐α was raised by 2.26‐fold in the LPS‐induced group in contrast to the control group (Figure 2D). IL‐1β and MCP‐1 mRNA expressions increased by 12.74‐fold (Figure 2E) and 3.1‐fold, respectively, in the LPS‐induced group compared with the control group (Figure 2F). When cells were pretreated with (R)‐salbutamol prior to LPS stimulation, the MCP‐1, IL‐1β and TNF‐α mRNA levels were markedly decreased. The mRNA expression of TNF‐α, IL‐1β and MCP‐1 increased by 2.28‐, 12.41‐ and 3.36‐fold, respectively, in ICI‐118551–treated cells in contrast to the control group. TNF‐α, IL‐1β and MCP‐1 mRNA expressions were not obviously different in ICI‐118551–treated cells in contrast to LPS‐induced RAW264.7 cells (Figure 2D‐F), suggesting that (R)‐salbutamol acts on the β_2_ adrenergic receptor and reduces the expression of these cytokines. These findings suggested that (R)‐salbutamol inhibited the expression of MCP‐1, IL‐1β and TNF‐α via the β_2_ adrenergic receptor at the transcriptional level, which in turn reduced MCP‐1, IL‐1β and TNF‐α protein levels in LPS‐induced macrophages.

### 3.3 Effects of (R)‐salbutamol and (S)‐salbutamol on NO and ROS production in RAW 264.7 cells

#### 3.3.1 (R)‐salbutamol decreases NO and ROS production in LPS‐induced RAW264.7 cells

**Figure 3:**
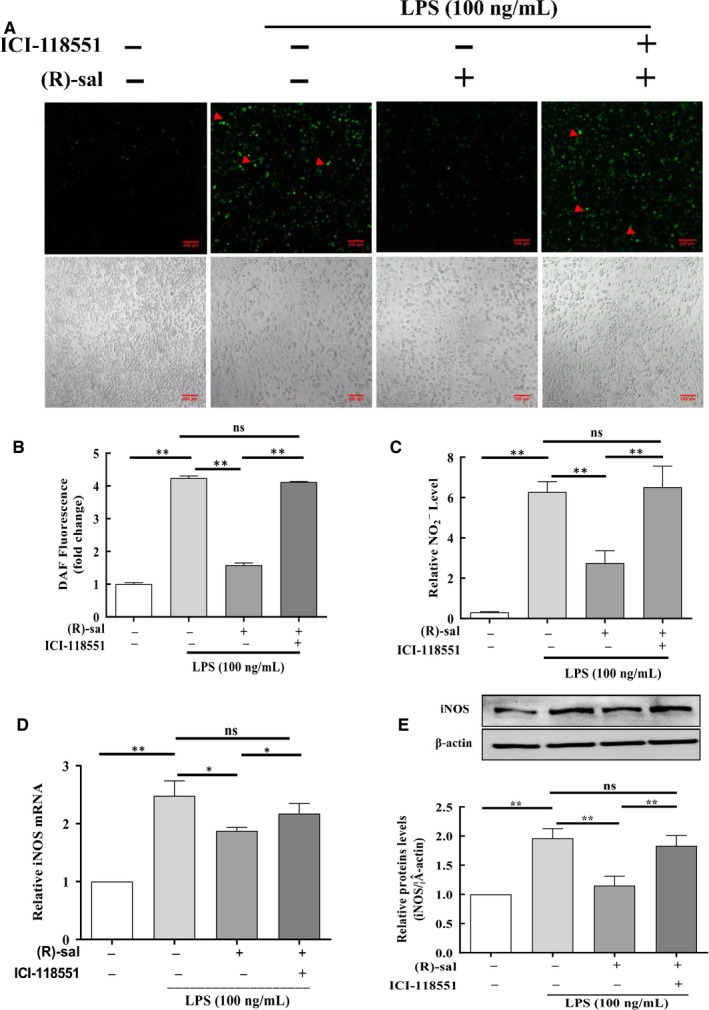
Effects of (R)‐salbutamol on LPS‐induced NO production and the expression of iNOS in RAW264.7 macrophages. A, Representative images of control cells, LPS‐induced cells, LPS‐induced cells pretreated with (R)‐salbutamol and LPS‐induced cells pretreated with ICI‐118551 labelled with the fluorescent NO indicator DAF‐FM DA (indicated by red arrows) for 30 min. Images were captured by a confocal microscope. B, Fluorescence quantification of NO was performed on digitalized images using ZEN 2011 Image Solution software (scale bars, 100 μm). C, Graph showing the content of NO_2_^−^ in the cell supernatants of control cells, LPS‐induced cells, LPS‐induced cells pretreated with (R)‐salbutamol and LPS‐induced cells pretreated with ICI‐118551 using Griess reagent. D,E, iNOS protein and mRNA expressions in control cells, LPS‐induced cells, LPS‐induced cells pretreated with (R)‐salbutamol and LPS‐induced cells pretreated with ICI‐118551 as quantified using Western blotting and qPCR. Data are presented as the mean ± SD. ^**^ *P* < .01, ^*^ *P* < .05; ns, not significant

Lipopolysaccharide can cause chronic inflammation, which is usually linked with higher NO levels.^37^ We hypothesized that (R)‐salbutamol exhibited anti‐inflammatory properties. To determine the anti‐inflammatory impact of (R)‐salbutamol on M1 macrophage polarization, the intracellular NO levels were determined using DAF‐FM DA, a NO^−^ sensitive fluorescence probe. Representative images revealed that the number of cells stained with DAF was increased (green) in LPS‐induced cells in contrast to control cells, whereas the cells pretreated with (R)‐salbutamol exhibited a decreased number of stained cells compared with control cells (Figure 3A). The level of DAF fluorescence increased by 424% in the LPS‐induced group compared with the control group, while the level of DAF fluorescence decreased by 2.68‐fold when cells were pretreated with (R)‐salbutamol (Figure 3B). Collectively, treatment with ICI‐118551 increased NO levels, suggesting that blocking the β_2_ adrenergic receptor could reduce the effects of (R)‐salbutamol on LPS‐induced RAW264.7 cells. In addition, the NO concentration in culture supernatant was quantified by examining the levels of nitrite (a stable NO breakdown product) using the Griess assay. Compared with control conditions, the expression of NO_2_^−^ increased by 20.25‐fold with LPS stimulation, and NO_2_^−^ expression decreased by 2.28‐fold with (R)‐salbutamol pretreatment (Figure 3C). M1 macrophages have also been shown to activate iNOS to produce NO from L‐arginine. To investigate whether (R)‐salbutamol could exert its effect on the level of iNOS, iNOS protein and mRNA levels were determined. When cells were induced with LPS, the iNOS mRNA level increased, and when cells were pretreated with (R)‐salbutamol, the iNOS mRNA level decreased (Figure 3D). Consistent with the mRNA results, the iNOS protein level was increased in cells induced with LPS and decreased in cells pretreated with (R)‐salbutamol (Figure 3E). Similarly, the iNOS protein and mRNA levels were raised when cells were exposed to ICI‐118551. In conclusion, our findings support the anti‐inflammatory properties of (R)‐salbutamol.

**Figure 4:**
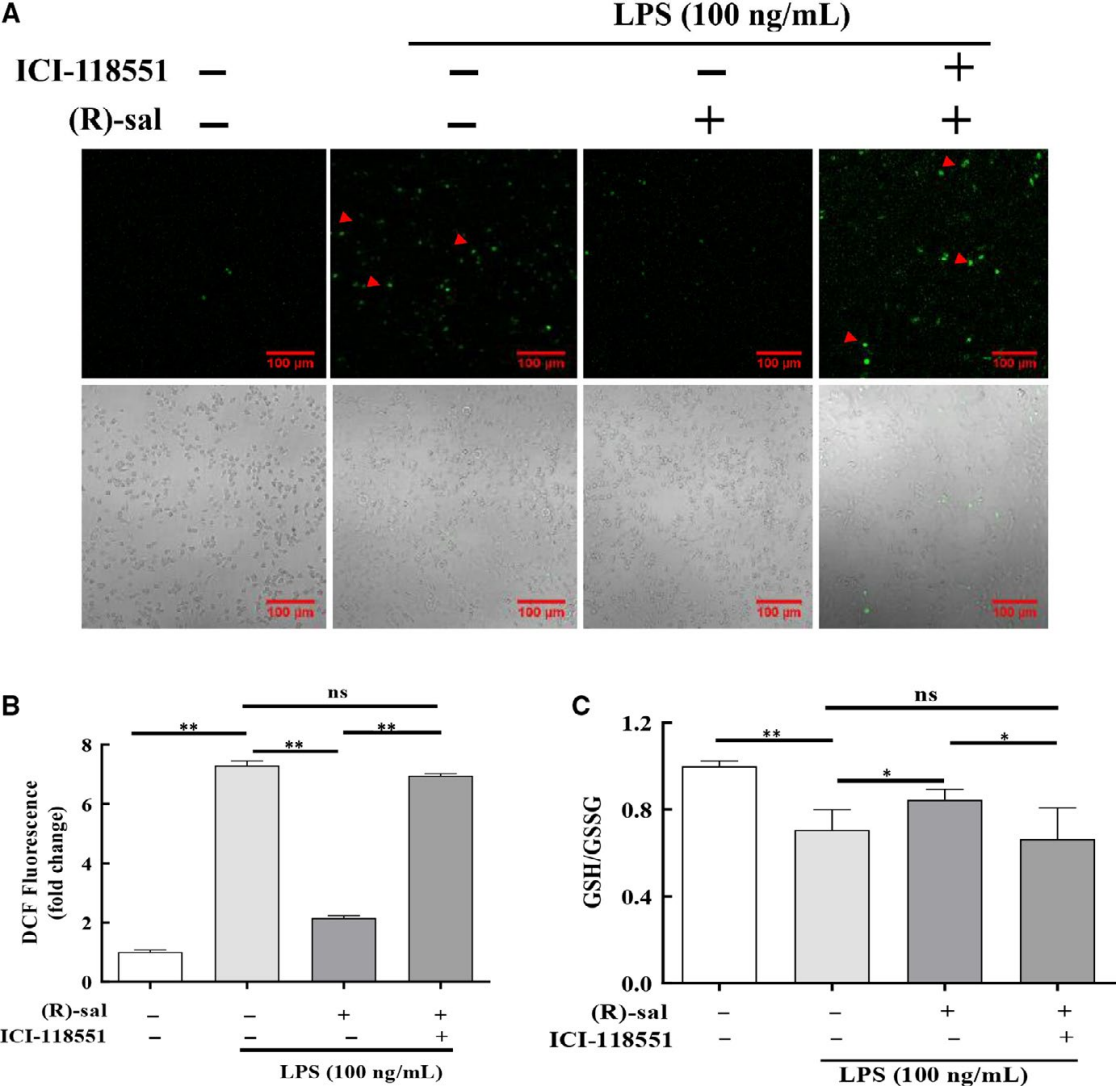
Effects of (R)‐salbutamol on the LPS‐induced production of ROS and GSH/GSSG in RAW264.7 macrophages. A, ROS formation (indicated by red arrows) of control cells, LPS‐induced cells, LPS‐induced cells pretreated with (R)‐salbutamol and LPS‐induced cells pretreated with ICI‐118551 was monitored with the fluorescence probe DCFH‐DA. B, Fluorescence quantification of ROS was performed on digitalized images using ZEN 2011 Image Solution software (scale bars, 100 μm). C, The GSH/GSSG ratio. Data are presented as the mean ± SD. ^**^ *P* < .01, ^*^ *P* < .05; ns, not significant

Lipopolysaccharide can promote cell apoptosis via mitochondrial dysfunction. Cellular ROS are primarily generated by mitochondria. ROS regulate various cell functions, such as apoptosis, cell survival and inflammation. In this study, ROS was visualized with the DCFH‐DA dye. The number of stained cells was lower in the (R)‐salbutamol pretreatment group in contrast to the cohort without (R)‐salbutamol pretreatment (Figure 4A). The level of DCF was increased to 7.30‐fold in LPS‐induced RAW264.7 cells as compared to control group, while pretreatment with (R)‐salbutamol decreased DCF by 3.38‐fold compared with LPS treatment group (Figure 4B) and iNOS levels at 12 hours, suggesting that β_2_ adrenergic receptor activation is required for M1 polarization.

#### 3.3.2 (S)‐salbutamol increases NO and ROS production in RAW264.7 cells

(S)‐enantiomer salbutamol was tested to study whether (S)‐salbutamol has similar effects as its (R)‐enantiomer. The level of DAF fluorescence, which was related to NO, was increased 6.48‐fold in cells pretreated with at 10 µmol/L in comparison with the control cells (Figure S3A‐B). The level of DCF fluorescence, which was related to ROS, was increased 6.53‐fold when cells were pretreated with (S)‐salbutamol at 10 µmol/L when contrasted to the control group (Figure S3C‐D).

Pretreatment with the (S)‐enantiomer of salbutamol increased the levels of both NO and ROS. This result was the opposite of the effects of the (R)‐enantiomer of salbutamol, which decreased the level of both NO and ROS in LPS‐induced cells. These results indicate that the (S)‐enantiomer of salbutamol may have different mechanisms than its (R)‐enantiomer in terms of the activation of macrophages in the inflammatory response.

### 3.4 (R)‐salbutamol increases the ratio of GSH/GSSG in LPS‐induced RAW264.7 cells

Since glutathione (GSH) is a direct scavenger for excessive ROS.^38^ The effect of (R)‐salbutamol on intracellular GSH was also evaluated in this study. Intracellular GSH/GSSG ratios decreased to 70.60% of control levels when cells were induced with LPS, and the ratio of GSH/GSSG increased by 84.50% in cells pretreated with (R)‐salbutamol compared with control (Figure 4C). Similarly, the ratio of GSH/GSSG was not changed in ICI‐118551‐treated cells compared with LPS‐induced RAW264.7 cells. These findings indicate that (R)‐salbutamol can increase the ratio of GSH/GSSG in LPS‐induced RAW264.7 cells.

### 3.5 (R)‐salbutamol rescues mitochondrial respiration and inhibits aerobic glycolysis in the LPS‐induced RAW264.7 cells

**Figure 5:**
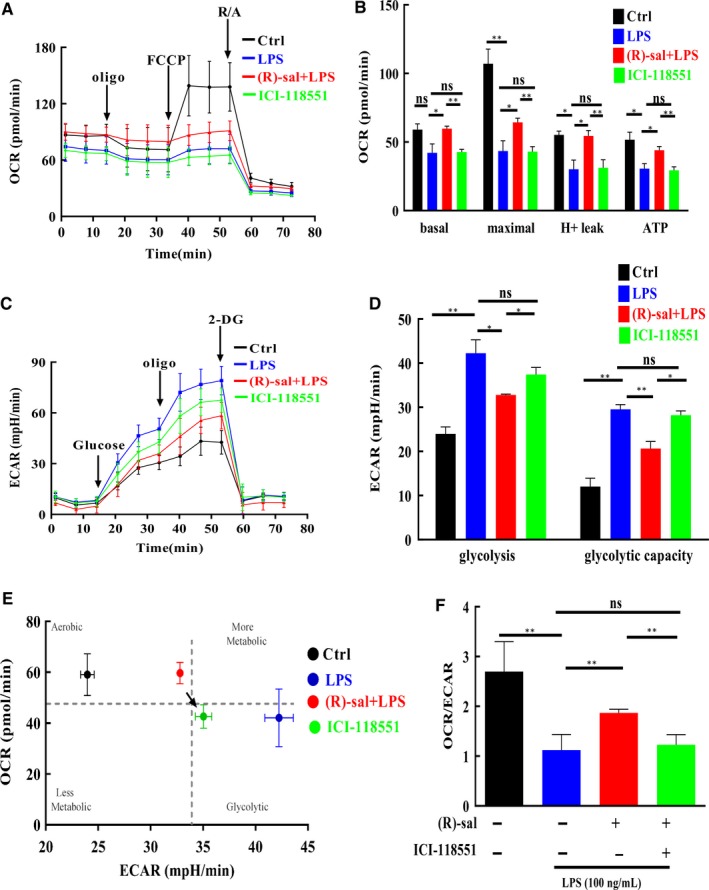
(R)‐salbutamol rescues mitochondrial respiration and inhibits Warburg metabolism (aerobic glycolysis) of LPS‐induced RAW264.7 cells. A, Mitochondrial stress test was carried out by the sequential addition of 0.5 µmol/L rotenone/antimycin A, 0.5 µmol/L FCCP and 1 µmol/L oligomycin. B, Maximal and basal respiration, H^+^ leak and ATP production (from left to right) are shown. C, Glycolysis stress test was carried out by sequential addition of 50 mmol/L 2‐DG, 1 µmol/L oligomycin and 10 mmol/L glucose. D, Glycolysis and glycolytic capacity are shown. E, Bioenergetic profiles obtained by plotting the maximal ECAR and OCR as quantified in (B) and (D). F, The ratio of OCR/ECAR. Oligo: oligomycin, an ATP synthase blocker; FCCP: carbonyl cyanide 4‐(trifluoromethoxy) phenylhydrazone; R/A: rotenone and antimycin A; 2‐DG: 2‐deoxyglucose. All data are shown as the mean ± SD. ^**^ *P* < .01, ^*^ *P* < .05; ns, not significant

Macrophage activation elicits changes in metabolic profiles according to activation state. It has been shown that LPS‐induced macrophages adopt glycolytic metabolic profiles.^39^ The impact of β_2_ adrenergic receptor activation on the LPS‐induced Warburg metabolism (aerobic glycolysis) of macrophages was investigated using measurements of the OCR and ECAR using an extracellular flux analyser. The OCR of LPS‐induced cells in response to (R)‐salbutamol over time was determined in a mitochondrial stress test (Figure 5A). We further analysed indices representing an alteration in mitochondrial respiration and found that basal and maximal respiration was reduced by 71.23% in the LPS‐induced group in contrast to the control group, which indicated some disruption of oxidative phosphorylation (OXPHOS). Basal and maximal respiration was increased in (R)‐salbutamol‐pretreated cells compared with LPS‐induced cells (Figure 5B). Post‐inclusion of the ATP synthase inhibitor oligomycin to the RAW264.7 cells, LPS induced a 59.29% decrease in the OCR, and (R)‐salbutamol induced a 69.59% increase in the OCR of LPS‐induced cells (Figure 5B). The difference between ATP production and basal respiration is considered an indirect measure of effective oxygen consumption during ATP synthesis (proton leak). The proton leak of LPS‐induced cells was decreased by 54.53% compared with that of control cells. Taken together, the bioenergetic profiles suggest that (R)‐salbutamol likely rescued OXPHOS failure in LPS‐induced RAW264.7 cells (Figure 5B). OCR levels were was not changed in LPS‐induced cells with the addition of ICI‐118551. The OCRs reported above were corrected by rotenone/antimycin A and were attributed to respiratory chain activity. Taken together, mitochondria‐independent OCR levels were markedly raised in macrophages exposed to LPS in comparison with those that were not. These findings were normalized to control levels by (R)‐salbutamol, suggesting that (R)‐salbutamol could be a potential protective compound against the LPS‐induced decrease in mitochondrial respiration via the β_2_ adrenergic receptor and that this mechanism caused the change in metabolic profiles.

In addition to the measurement of mitochondrial respiration, ECAR, an indirect measurement of the cellular glycolytic rate, was also determined (Figure 5C). Compared with control conditions, LPS stimulation upregulated aerobic glycolysis, while (R)‐salbutamol inhibited aerobic glycolysis (Figure 5D). The difference between basal and maximal ECAR rates is known as the glycolytic reserve capacity. LPS‐induced cells exhibited higher glycolytic reserve capacity than control cells, suggesting that LPS‐induced cells exhibited high metabolic plasticity to maintain intracellular ATP content (Figure 5D). Collectively, these data suggested that (R)‐salbutamol likely mediated the metabolism shift in LPS‐induced cells and that it protected against LPS‐induced inflammation. An in‐depth investigation is needed to clarify the intrinsic mechanism as well as to identify the molecular target of (R)‐salbutamol in the process of LPS‐induced inflammation.

To obtain an improved overall understanding of the bioenergetic profiles of (R)‐salbutamol in LPS‐induced cells, basal ECAR was plotted against mitochondrial OCR (Figure 5E). Two distinct groups of cellular bioenergetic profiles were identified. The control cells and LPS‐induced cells pretreated with (R)‐salbutamol had a more aerobic phenotype than LPS‐induced cells and ICI‐118551‐treated cells, which were more glycolytic (Figure 5E). The ratio of OCR/ECAR was reduced in LPS‐induced cells compared with control cells, whereas the ratio of OCR/ECAR was raised when LPS‐induced cells were pretreated with (R)‐salbutamol (Figure 5F). Similarly, ICI‐118551 treatment did not change the ratio of OCR/ECAR in LPS‐induced cells. Collectively, cells pretreated with (R)‐salbutamol displayed lower glycolytic capacities than those with LPS‐induced cells, indicating that (R)‐salbutamol can restore the maximal glycolytic and respiratory capacities to near‐normal levels.

### 3.6 Metabolomics of (R)‐salbutamol in LPS‐induced RAW264.7 cells

#### 3.6.1 Method validation

To investigate the metabolic mechanisms of (R)‐salbutamol, an untargeted cell metabolomics approach was employed. To shorten the analytical time and increase the number of metabolic that could be identified in this study, we simultaneously performed analysis in two fractions: organic (lipophilic) extract and aqueous (hydrophilic) extract. To ensure the stability, precision and repeatability of the UHPLC/ESI‐TIMS TOF‐MS/MS method, this study duplicated analytical QC samples and analysed set of parallel samples with identical methods. The analytical method was validated by extracting the ion chromatographic peaks of five ions in positive ion mode (with RTs and m/z pairs of 0.48‐306.0764, 1.2‐120.0807, 2.66‐364.8754, 6.23‐278.2114 and 6.23‐278.2114) and five ions in negative ion mode (RTs and m/z pairs of 0.49‐306.0764, 3.79‐243.1711, 11.73‐500.2772, 12‐526.2925 and 12.39‐478.2925) were selected. The relative standard deviation (RSD) of the RTs reflecting the repeatability, injection precision and system stability was estimated to be 0.48%‐12.39%, 0.03%‐1.14% and 0.48%‐12.40%, respectively, while the RSDs of peak area were within the ranges of 2.45%‐13.95%, 1.51%‐7.92% and 1.69%‐8.79% for repeatability, injection precision and system stability, respectively (Table S2). These findings demonstrated that the present analytical method is appropriate for metabolomics analysis, as the data showed great stability and reproducibility.

#### 3.6.2 Multivariate analysis and identification of potential biomarkers

**Figure 6:**
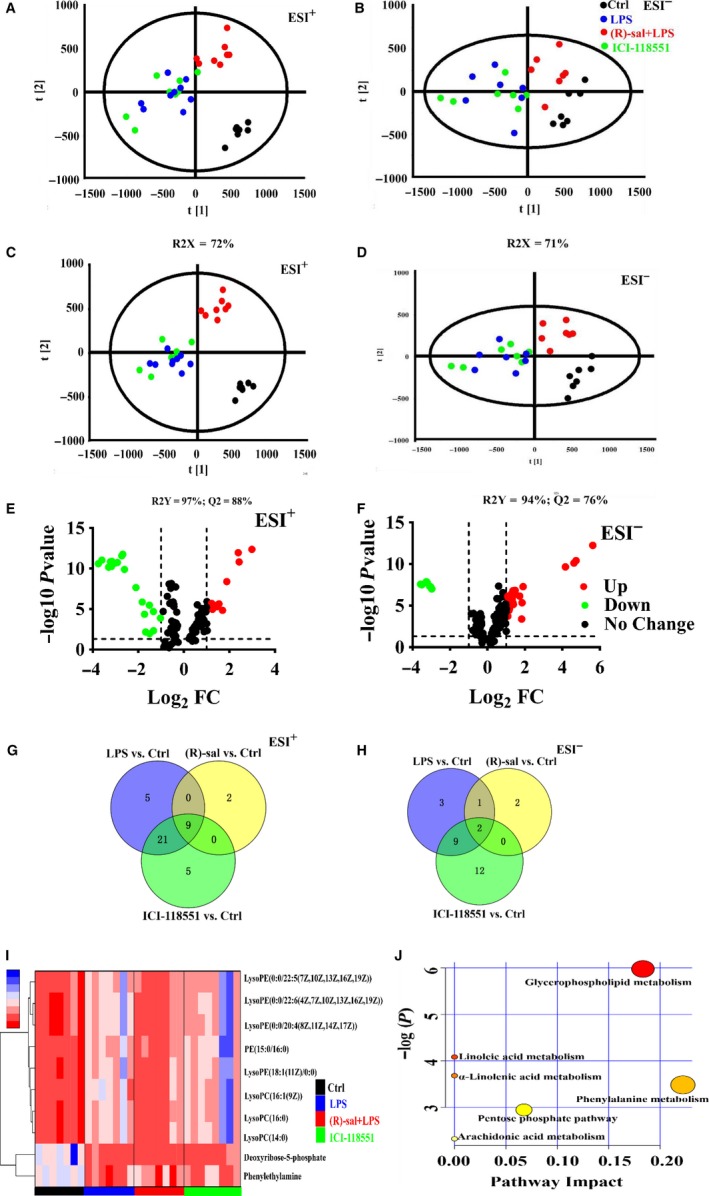
Effects of (R)‐salbutamol on the metabolomics of LPS‐induced RAW264.7 macrophages. A,B, PCA score plot of macrophages in the control, LPS‐induced, (R)‐salbutamol and ICI‐118551 cohorts in the (A) ESI (+) and (B) ESI (−) modes. C,D, PLS‐DA score plot of the control, LPS‐induced, (R)‐salbutamol and ICI‐118551 groups based on the macrophage metabolic profiles in the (C) ESI (+) and (D) ESI (−) modes. E,F, Volcano plots of p values and fold change in the (E) ESI (+) and (F) ESI (−) modes. G,H, Venn diagrams of increased (G) or decreased metabolites (H) found in the binary comparisons of LPS vs control, (R)‐salbutamol vs control and ICI‐118551 vs control corresponding to the numbers reported in Table S3. I, Unsupervised hierarchical clustering of heat map changes in potential biomarker content in the control, LPS, (R)‐salbutamol and ICI‐118551 groups in the ESI (+) and ESI (−) modes. Columns: samples; Rows: biomarkers. Metabolite content—blue: high metabolic content; red: low metabolic content (J) Pathway analysis of the differential metabolism in the control, LPS, (R)‐salbutamol and ICI‐118551 groups based on topology analysis (x‐axis) and enrichment analysis (y‐axis) scores. The size and colour of each circle represent the impact factor of each pathway as well as the *P* value. Red pathways were more affected. This analysis was done via MetaboAnalyst 4.0. All data are shown as the mean ± SD. ^**^ *P* < .01, ^*^ *P* < .05; ns, not significant

Powerful statistical modelling tools, such as the TIMS TOF‐MS/MS analysis platform and Progenesis QI software in combination with PCA, can provide insights into the differences among experimental groups. A robust TIMS TOF‐MS/MS analysis platform was used to ascertain likely molecular markers with molecular weights and MS/MS spectra. Progenesis QI software was employed to search biochemical databases, and the PCA method was employed to perform an unsupervised pattern recognition method. The PCA score plot of the data in positive and negative ionization mode showed that the LPS and ICI‐118551 groups overlapped but clustered differently from the control and (R)‐salbutamol groups, in the direction of the first principal component (R2X = 72% in positive mode, R2X = 71% in negative mode) (Figure 6A‐B), which suggests that the endogenous metabolite profiles were significantly different from those of the LPS‐induced group. Additionally, the metabolite profiles of the LPS‐induced group and the (R)‐salbutamol group clustered separately, suggesting that R‐salbutamol altered the metabolites of LPS‐induced cells.

To determine differences between the clusters, PLS‐DA and OPLS‐DA were used to amplify discrimination and identify the metabolite differences among these groups. The PLS‐DA showed that the cells pretreated with (R)‐salbutamol had a high cumulative predictive capacity (Q2 = 0.88 in positive mode, Q2 = 0.94 in negative mode) and was well‐represented in the data (R2Y = 0.88 in positive mode, R2Y = 0.76 in negative mode) (Figure 6C‐D). These findings suggested that 10 µmol/L (R)‐salbutamol could alter the metabolome of LPS‐induced RAW264.7 cells. In addition, our data showed that the metabolite profiles of the LPS group and ICI‐118551 group overlapped (Figure 6A‐D), suggesting that ICI‐118551 abrogated the inhibitory properties of (R)‐salbutamol.

To compare metabolite changes among these samples, volcano plots were constructed, and the data revealed metabolites that were significantly upregulated (red plots) and downregulated (green plots) (Figure 6E‐F). In addition, Venn diagrams were constructed to compare the characteristics of each metabolite in the LPS‐induced group, (R)‐salbutamol–treated group and ICI‐118551–treated group (Figure 6G‐H). Based on VIP values > 1, *P* < .05 and fold change > 2, a total of 11 potential biomarkers were ascertained from the peak profile of metabolomics by HMDB, EZinfo software and LipidMAPS (Table S3). These identified metabolites were deoxyribose‐5‐phosphate, lysophosphatidylcholine (LysoPC) (14:0), LysoPC (16:1(9Z)), lysophosphatidylethanolamine (LysoPE) (0:0/22:6(4Z,7Z,10Z,13Z,16Z,19Z)), LysoPE (0:0/20:4(8Z,11Z,14Z,17Z)), LysoPE (0:0/22:5(7Z,10Z,13Z,16Z,19Z)), LysoPC (16:0), LysoPE (18:1(11Z)/0:0), phenylethylamine and phosphatidylethanolamine (PE) (15:0/16:0). Among those metabolites, phenylethylamine and deoxyribose 5‐phosphate were decreased in all the LPS‐induced group, (R(R)‐salbutamol–treated group and ICI‐118551–treated group in contrast to the control group. The following metabolites were markedly raised in the LPS‐induced group in contrast to the control group: LysoPC (14:0), LysoPC (16:1(9Z)), LysoPE (0:0/22:6(4Z,7Z,10Z,13Z,16Z,19Z)), LysoPE (0:0/20:4(8Z,11Z,14Z,17Z)), LysoPE (0:0/22:5(7Z,10Z,13Z,16Z,19Z)), LysoPC (16:0), LysoPE (18:1(11Z)/0:0) and PE (15:0/16:0). Additionally, LysoPC (14:0), LysoPC (16:1(9Z)), LysoPE (0:0/22:6(4Z,7Z,10Z,13Z,16Z,19Z)), LysoPE (0:0/20:4(8Z,11Z,14Z,17Z)), LysoPE (0:0/22:5(7Z,10Z,13Z,16Z,19Z)), LysoPC (16:0), LysoPE (18:1(11Z)/0:0) and PE (15:0/16:0) were notably increased in the (R)‐salbutamol–treated group compared with the LPS‐induced group, and ICI‐118551 treatment inhibited the impact of (R)‐salbutamol treatment (Table S3). A heat map of the unsupervised hierarchical clustering was constructed to visualize the changes in the contents of potential biomarkers. A heat map (colour changes from red to blue) indicated the downregulated and upregulated metabolites among the groups (Figure 6I). Based on the colour distribution, cells pretreated with (R)‐salbutamol were more similar to the control group than to the LPS‐induced group. Our data showed different intensities of various identical metabolites in various samples from the different groups (Figure S4), suggesting that (R)‐salbutamol might effectively alter the metabolic pattern of LPS‐induced cells.

#### 3.6.3 Metabolic pathways

To further elucidate the metabolic pathways that were regulated by (R)‐salbutamol in LPS‐induced cells, the above‐mentioned biomarkers were further analysed using MetaboAnalyst 4.0. Several pathways, including glycerophospholipid metabolism, phenylalanine metabolism and the pentose phosphate pathway, were highly impacted, suggesting that these pathways are involved in the (R)‐salbutamol–mediated M1 polarization of LPS‐induced cells (Figure 6J). In particular, glycerophospholipid metabolism was the most highly impacted pathway, suggesting that glycerophospholipid metabolism, but not phenylalanine metabolism and the pentose phosphate pathway, is likely involved in (R)‐salbutamol–mediated M1 polarization. Taken together, our findings suggested that the gradual variation in effects was due to perturbations of endogenous metabolites in macrophages under different conditions.

## 4 DISCUSSION

This study investigates the effects of (R)‐salbutamol, a β_2_ receptor agonist, on M1 macrophage polarization and metabolic alterations in LPS‐induced RAW264.7 cells. β_2_ receptor agonists are the cornerstone bronchodilating agents used to treat obstructive lung diseases.^15^ These agents have also been demonstrated to possess anti‐inflammatory properties on airways and may reduce pro‐inflammatory mediators as well as prevent tissue oedema and exudate.^40^, ^41^ A commonly used β_2_ receptor agonist is racemic salbutamol which contains both (R)‐salbutamol and (S)‐salbutamol. Racemic salbutamol reduces carrageenan‐induced paw oedema in rodents^42^ via a β_2_ receptor‐dependent mechanism.^43^ On the other hand, studies showed that (S)‐salbutamol likely exacerbates asthma^18^ and results in pro‐inflammatory influences.^44^ In this study, we demonstrated that the (S)‐enantiomer of salbutamol differs from the (R)‐enantiomer. (S)‐salbutamol increases NO and ROS levels in macrophages instead of inhibiting these molecules like its counterpart. This finding suggests that the (S)‐enantiomer of salbutamol may play different roles in macrophage polarization in the inflammatory response. The mechanism of the differences between the salbutamol (S)‐ and (R)‐enantiomers needs further investigation. Collectively, we found that (R)‐salbutamol inhibited the LPS‐induced M1 phenotype of macrophages, which may be associated with the anti‐inflammatory mechanism of (R)‐salbutamol.

Macrophages are crucial in host defence against infections. Inflammatory diseases and cancer have been documented to possess an excess of pro‐inflammatory molecules such as IL‐1β, TNF‐α, NO and ROS. Inflammation is a double‐edged sword. On one hand, it is responsible for stimulating tissue regrowth and halting worsening cellular injury. However, prolonged and uncontrolled inflammatory responses lead to severe tissue damage culminating with multi‐organ failure with high mortality rates. The present study showed that 10 µmol/L (R)‐salbutamol could reduce the expression of typical cytokines found in M1 macrophages (ie MCP‐1, IL‐1β and TNF‐α) more effectively than 100 nmol/L salbutamol.^43^ These results mirror those of Tanaka et al, who found that salbutamol exhibited protective anti‐inflammatory effects on LPS‐treated rat peritoneal macrophages.^16^ Additionally, we found that (R)‐salbutamol reduced the production of NO, iNOS and ROS (Figure 3). NO works at almost all stages of inflammation by regulating of inflammatory cell transmission.^45^ In LPS‐induced inflammation, NO is produced by iNOS. Racemic salbutamol reportedly inhibited the mRNA and protein levels of iNOS via the ERK pathway in rat peritoneal macrophages.^46^ Similarly, both exogenous and endogenous ROS cause oxidative DNA damage that alters cell signal transduction, a deleterious process that is observed in several stages of tumorigenesis such as tumour development and progression. ROS production is amplified in cells exposed to LPS. Our data showed that (R)‐salbutamol can prevent excessive ROS through the LPS‐mediated macrophage pro‐inflammatory response. Furthermore, the inhibitory properties of (R)‐salbutamol could be blocked by specific β_2_ receptor antagonists, ICI‐118551. In addition, the ratio of GSH/GSSG was increased in LPS‐induced cells pretreated with (R)‐salbutamol. GSH is a cytosol sulfhydryl antioxidant and can scavenge excessive ROS, which results in the reduction of intracellular ROS in LPS‐induced macrophages. This reduction in oxidative stress inhibits manufacturing of pro‐inflammatory cytokines, such as MCP‐1, IL‐1β and TNF‐α.^47^ In summary, we discovered that (R)‐salbutamol, the (R)‐enantiomer of a widely prescribed β_2_ receptor agonist, may be critical in the LPS‐induced switch of RAW264.7 cells to the M1 phenotype via the β_2_ adrenergic receptor. Nevertheless, its effects on M2 macrophage polarization is less clear, requiring further investigation.

Cell metabolism reprogramming is essential for the inflammatory process, especially during macrophage polarization.^48^ Immune cell activation depends heavily on intracellular glucose metabolism.^49^ In this study, we investigated the bioenergetic profiles of mitochondrial respiration and aerobic glycolysis in LPS‐induced cells and compared them with cells pretreated with (R)‐salbutamol and ICI‐118551. We found that (R)‐salbutamol significantly inhibited intracellular aerobic glycolysis. Thus, these data revealed that (R)‐salbutamol rescued basal respiration and reduced OCR, respiratory reserves, ATP production and maximal respiration in LPS‐induced cells, suggesting that LPS markedly altered cellular metabolism. In the presence of oxygen, the metabolic phenotype was characterized by the production of glycolytic energy, which is highly similar to the Warburg effect seen in tumour cells.^50^ Our data suggested that LPS stimulation leads to metabolic reprogramming *via* switching OXPHOS to aerobic glycolysis. These results are similar to reports demonstrating a decrease in bone marrow‐derived macrophage glycolysis after pretreatment with racemic salbutamol.^51^ LPS could disrupt mitochondrial homeostasis by enhancing aerobic glycolysis accompanied, and this was accompanied by a decrease in mitochondrial respiration. In this study, we found that cells pretreated with (R)‐salbutamol could reverse this phenotype by normalizing its metabolic manner. However, we should consider the underlying mechanism of how (R)‐salbutamol inhibits the aerobic effect facilitated by LPS. The Warburg effect suggests that cells under severe oxidative stress benefit from transitioning from oxidative to reductive metabolism. Taken together, further studies are needed to investigate how (R)‐salbutamol downregulates aerobic glycolysis and how this enhances mitochondrial respiration.

Previous studies showed that metabolic reprogramming is critical for the maturation and polarization of immune cells.^52^ In combination with multivariate statistical analysis methods, we identified metabolites that define the M1 phenotype of polarized macrophages. PCA and PLS‐DA revealed that there are marked differences in the metabolic profiles of the LPS‐induced, (R)‐salbutamol–treated, ICI‐118551–treated and control groups. The metabolic profiles of cells pretreated with (R)‐salbutamol were close to those exhibited by the control group. These data further consolidated our findings and suggest that (R)‐salbutamol regulates the metabolites of LPS‐induced cells. The present untargeted metabolomics data identified, for the first time, 11 potential biomarkers associated with metabolic changes in LPS‐induced cells pretreated with (R)‐salbutamol. Glycerophospholipids were the most highly impacted metabolite and represent the main metabolic pathway that regulated M1 polarization in this study. Glycerophospholipids are major constituents of the cell membrane and lipoproteins that regulate cell metabolism and signalling in inflammation and cell differentiation.^53^ The upregulation of glycerophospholipids could be due to damage to the cell membrane. Phosphatidylcholines and phosphatidylethanolamines are the major glycerophospholipids in phospholipid membranes. LysoPCs are generated from the hydrolysis of phosphatidylcholines. LysoPCs could induce proinflammation through the upregulation of adhesion molecules and endothelial permeability.^54^, ^55^ Recent studies revealed that LysoPCs promote and stabilize a strong M1 phenotype during macrophage polarization^56^ and thereby increase ROS and NO production.^57^, ^58^, ^59^ The levels of PE and LysoPE increased in activated human macrophages.^60^ The present study showed that LysoPCs, PE and LysoPE levels were augmented in the LPS‐induced group. Pretreatment with (R)‐salbutamol downregulated LysoPCs, PE and LysoPE, suggesting that (R)‐salbutamol exerted anti‐inflammatory effects on LPS‐induced cells via regulating glycerophospholipid metabolism. Furthermore, pretreatment with (R)‐salbutamol favourably impacted RAW264.7 cells induced with LPS, as (R)‐salbutamol showed anti‐inflammatory efficacy by restoring the biomarkers identified in this study. Although this study aimed to understand how (R)‐salbutamol acts on LPS‐induced cells, pathways and/or signalling molecules, the mechanisms of racemic salbutamol and (S)‐salbutamol remain a question that warrants further investigation. Additionally, further studies evaluating the metabolic flux after LPS exposure and the effect of (R)‐salbutamol in this scenario could be helpful in identifying important pathways involved in regulating inflammatory processes.

**Figure 7:**
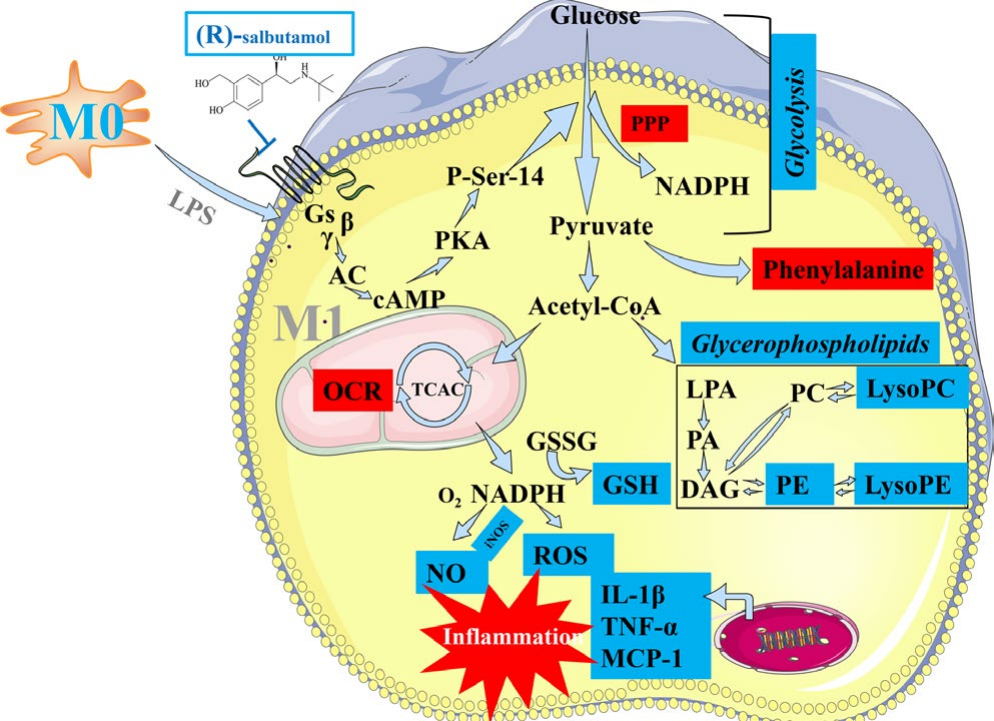
Potential inhibitory effects of (R)‐salbutamol on M1 macrophage polarization and metabolism. Red and blue boxes represent the activation and inhibition of some indices that were evaluated in this study. Words in black are metabolites that were not detected in this experiment. Italic words in the blue boxes represent the pathways that were most affected in this study

In conclusion, we found that (R)‐salbutamol blocked aerobic glycolysis, downregulated glycerophospholipid metabolism and alleviated LPS‐induced macrophage polarization, protecting against the subsequent pro‐inflammatory response in macrophage cells (Figure 7). These findings suggest that (R)‐salbutamol may be the major pharmacologically active component of racemic salbutamol and propose (R)‐salbutamol as a promising candidate drug for the treatment of inflammatory diseases. However, future studies are required to investigate upstream and downstream signalling molecules and elucidate the mechanism between the LPS and β2 receptor agonist pathways. These data may provide a new insight into the medicinal value of (R)‐salbutamol for inflammatory diseases treatment such as arthritis and CVD and propose it as (R)‐isomer in order to gain more positive outcomes during inflammation therapy.

## CONFLICT OF INTERESTS

The authors have declared no conflict of interest.

## AUTHORS’ CONTRIBUTIONS

Shangping Wang and Wen Tan designed the work; Shangping Wang, Fei Liu and Keai Sinn Tan carried out the experiments and analysed the data with the guidance of Wen Tan. Shangping Wang, Keai Sinn Tan, Hooi Leng Ser, Loh Teng Hern Tan and Learn‐Han Lee prepared the manuscript. All authors drafted or critically revised the manuscript for important intellectual content and approved the final version of the manuscript.

## Data Availability

The data sets used and/or analysed in this study are available from the corresponding author on reasonable request.
