# Supplementary figures and images for: Effect of (R)‐salbutamol on the switch of phenotype and metabolic pattern in LPS‐induced macrophage cells
Source: J Cell Mol Med. 2019 Nov 3;24(1):722–36. doi: 10.1111/jcmm.14780 (PMC6933346; doi:10.1111/jcmm.14780)

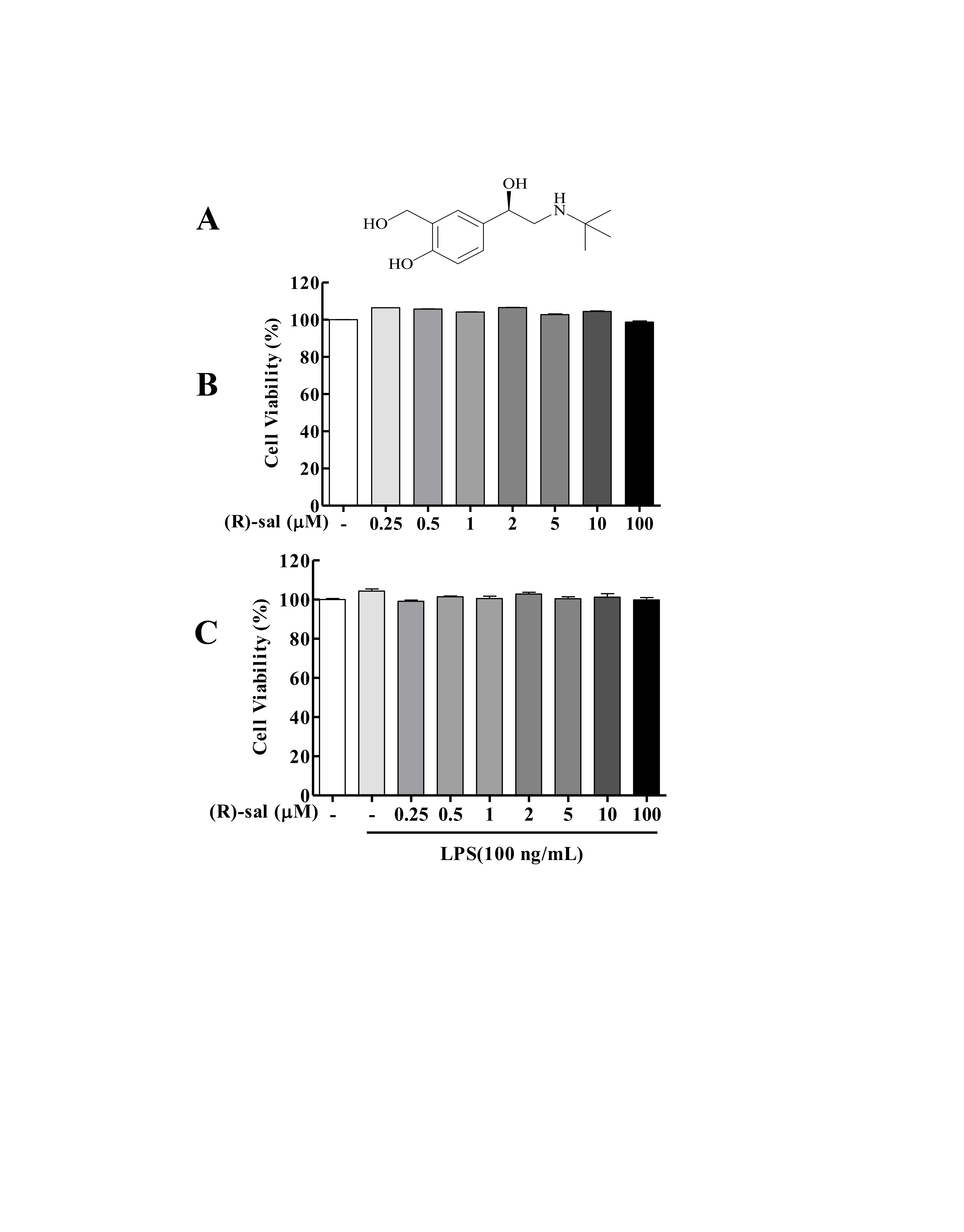

Supplement: Supplementary file 1 [file JCMM-24-722-s001.tif]

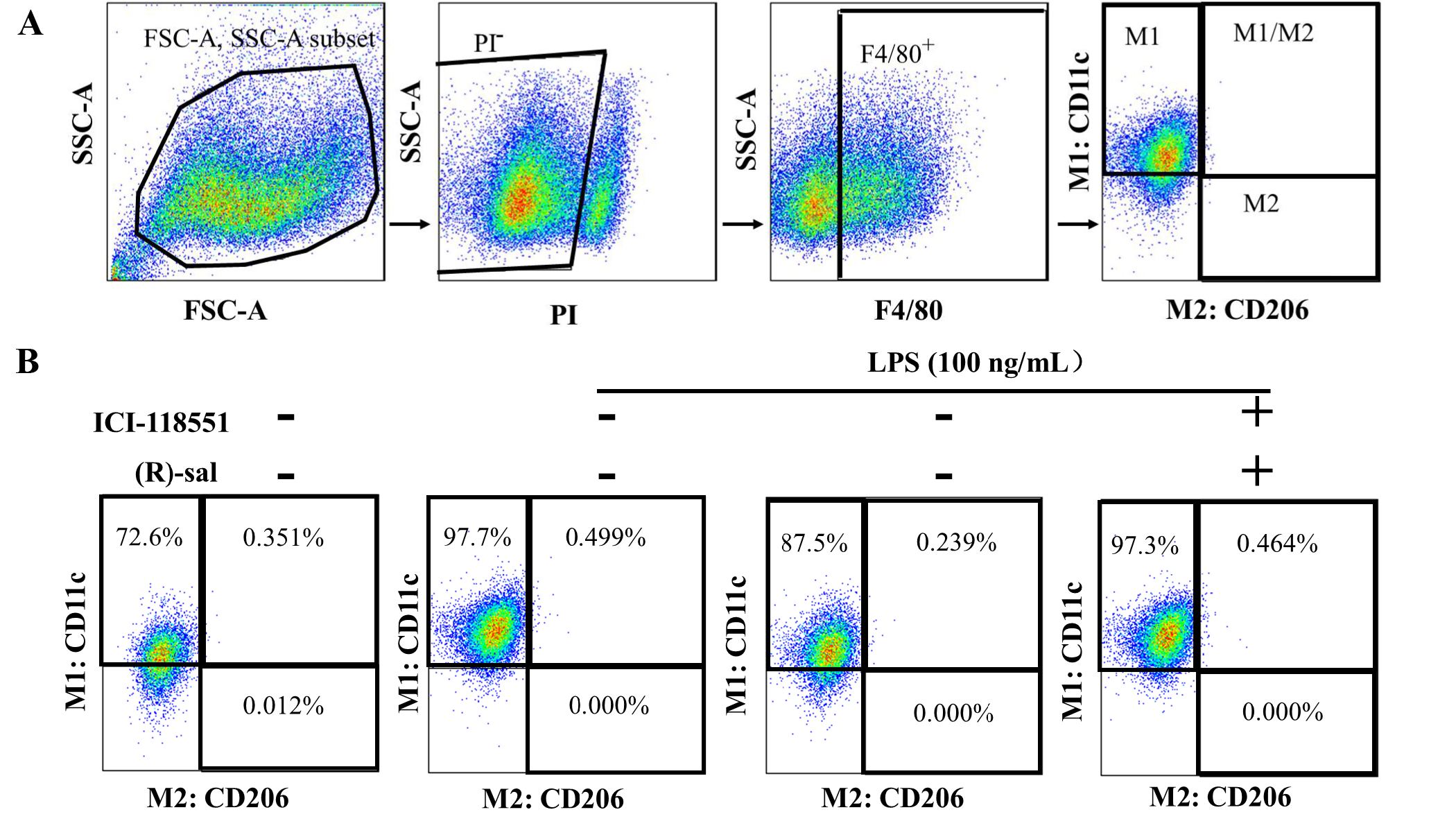

Supplement: Supplementary file 2 [file JCMM-24-722-s002.tif]

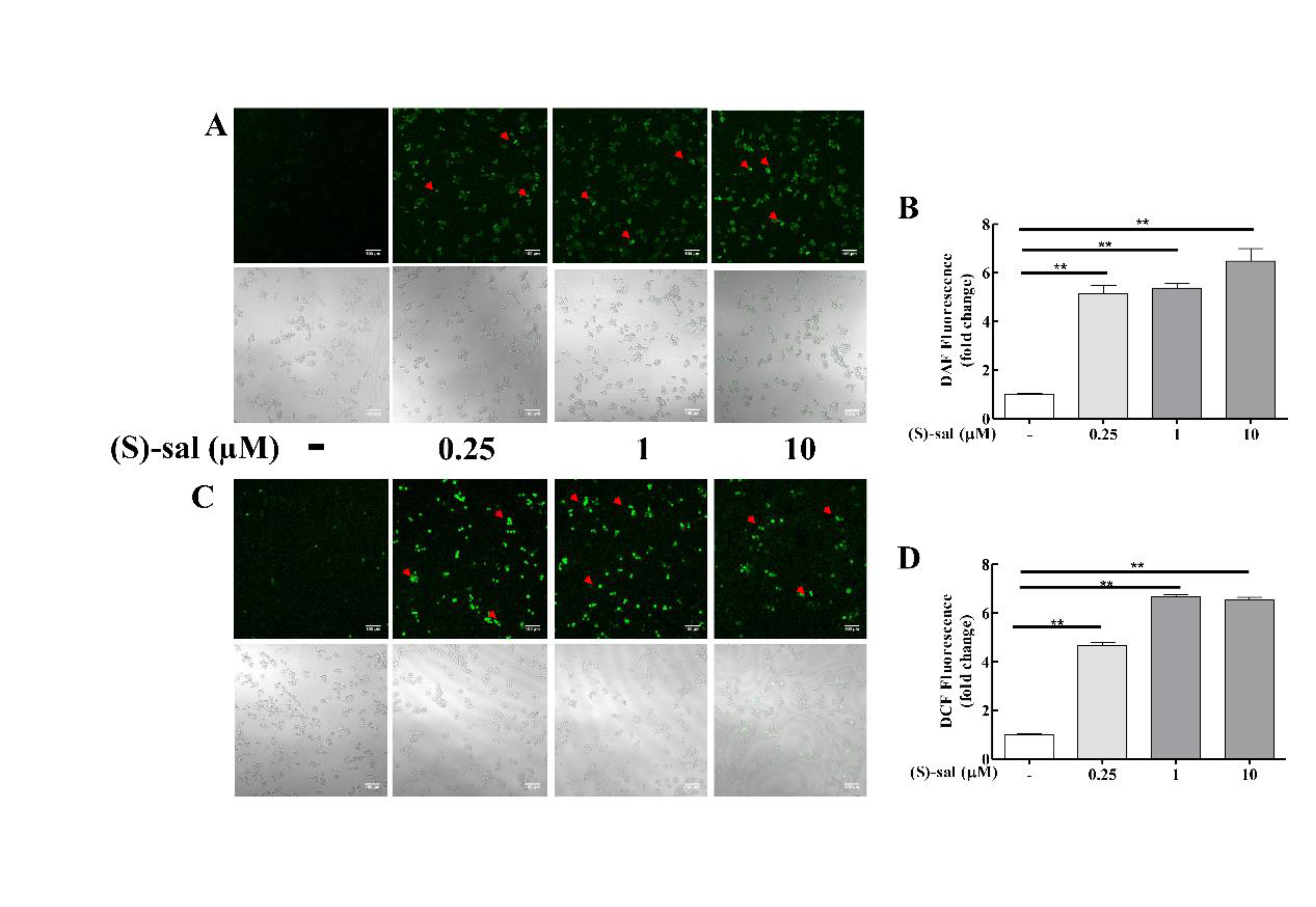

Supplement: Supplementary file 3 [file JCMM-24-722-s003.tif]

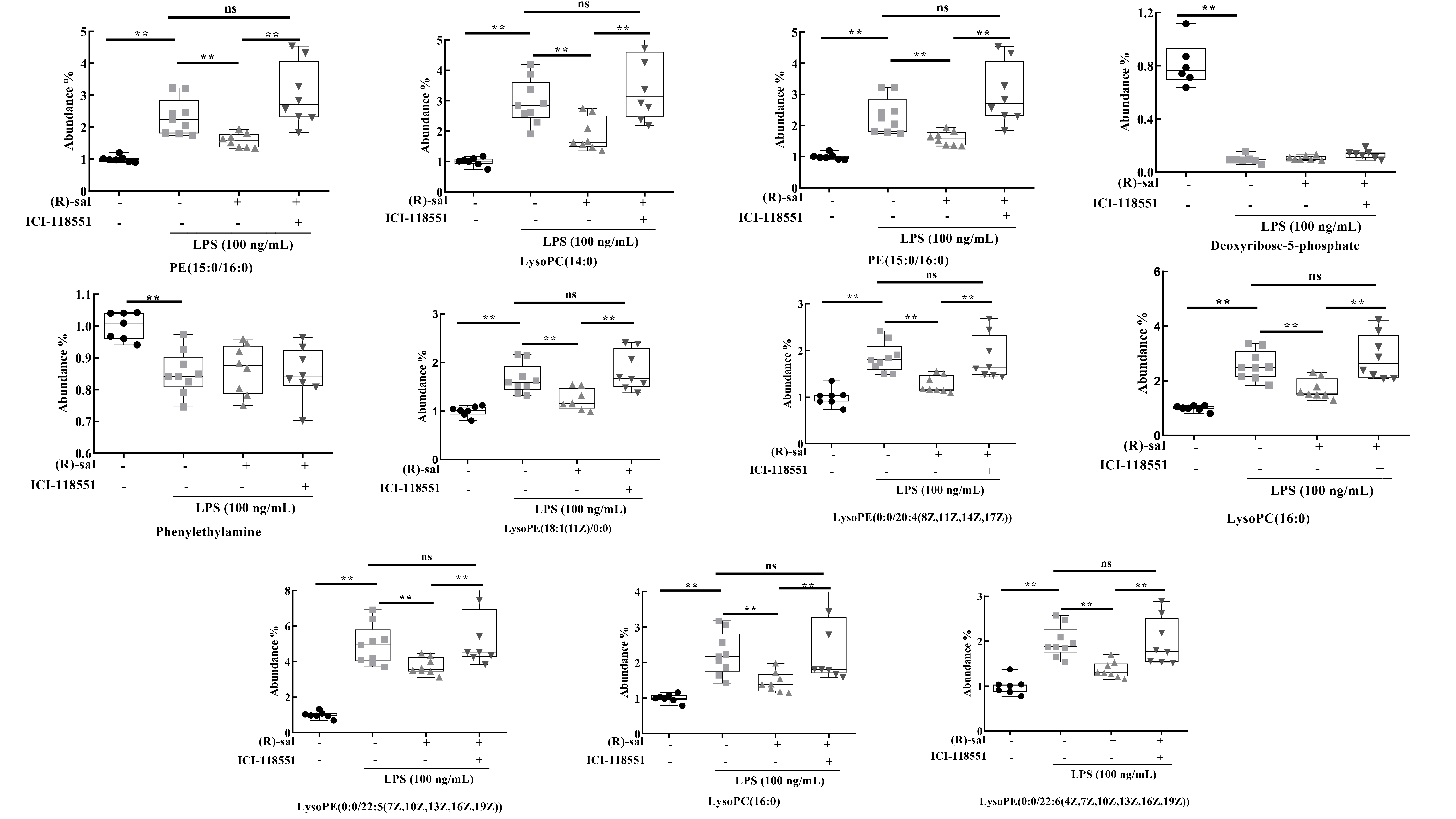

Supplement: Supplementary file 4 [file JCMM-24-722-s004.tif]
