# Supplementary material for: Effect of (R)‐salbutamol on the switch of phenotype and metabolic pattern in LPS‐induced macrophage cells
Source: J Cell Mol Med. 2019 Nov 3;24(1):722–36. doi: 10.1111/jcmm.14780 (PMC6933346; doi:10.1111/jcmm.14780)
Supplement: Supplementary file 5 [file JCMM-24-722-s005.docx]

**SUPPLEMENTARY TABLES**

**SUPPLEMENTARY TABLE 1**Primer sequences, sense and antisense, used in real-time PCR analysis. Abbreviations: MCP-1, monocyte chemotactic protein-1; NOS, inducible nitric oxide synthase; TNF-α, tumor necrosis factor-alpha; IL-1β, interleukin-1β; β-actin, beta-actin.

| **Primers** | **Sequence** |
| --- | --- |
| MCP-1 F | 5´-TCCCAATGAGTAGGCTGGAG-3´ |
| MCP-1 R | 5´-AAGTGCTTGAGGTGGTTGTG-3´ |
| IL-1β F | 5´-CTTCAGGCAGGCAGTATCACTC-3´ |
| IL-1β R | 5´-TGCAGTTGTCTAATGGGAACGT-3´ |
| TNF-α F | 5´-CGAGTGACAAGCCTGTAGCCC-3´ |
| TNF-α R | 5´-GTCTTTGAGATCCATGCCGTTG-3´ |
| iNOS F | 5´- CAACATCAGGTCGGCCATCACT-3´ |
| iNOS R | 5´- ACCAGAGGCAGCACATCAAAGC-3´ |
| β-actin F | 5´-TGCTGTCCCTGTATGCCTCT-3´ |
| β-actin R | 5´-TTTGATGTCACGCACGATTT-3´ |

**SUPPLEMENTARY TABLE 2**QC samples-based investigation of the analytical methods (accuracy (<10%), repeatability (<15%) and stability (<20%)). A total of five ions in positive ion mode and five ions in negative ion mode from the extracted ion chromatographic peaks were selected for method validation. The RSDs of retention time for injection precision, repeatability and system stability were estimated.

| Scan polarity | Ion pair | **Precision** | | | | **Repeatability** | | | | **System Stability** | | | | |
| --- | --- | --- | --- | --- | --- | --- | --- | --- | --- | --- | --- | --- | --- | --- |
|  |  | RT(min) | | Area | | RT(min) | | Area | | RT(min) | | | Area | |
|  |  | X | RSD% | X | RSD% | X | RSD% | X | RSD% | X | RSD% | X | | RSD% |
| ESI(+) | 0.48-306.0764 | 0.48 | 1.10 | 259104.00 | 5.29 | 0.48 | 1.56 | 250688.50 | 5.08 | 0.49 | 1.10 | 278926.90 | | 5.65 |
|  | 1.2-120.0807 | 1.20 | 0.63 | 796389.57 | 2.27 | 1.2 | 0.53 | 810861.40 | 9.40 | 1.2 | 0.68 | 927410.70 | | 4.18 |
|  | 2.66-364.8754 | 2.67 | 0.26 | 224280.57 | 7.92 | 2.66 | 0.19 | 230808.00 | 13.04 | 2.67 | 0.26 | 232354.60 | | 8.79 |
|  | 6.23-278.2114 | 6.24 | 0.08 | 1386516.00 | 1.48 | 6.23 | 0.08 | 1428493.00 | 7.43 | 6.23 | 0.06 | 1495514.00 | | 1.69 |
|  | 12.34-496.3393 | 12.35 | 0.04 | 311182.29 | 6.70 | 12.34 | 0.06 | 288162.70 | 9.67 | 12.34 | 0.04 | 270931.10 | | 4.36 |
| ESI(-) | 0.49-306.0764 | 0.49 | 1.10 | 230557.72 | 1.51 | 0.48 | 1.58 | 243716.01 | 2.45 | 0.48 | 1.10 | 264453.10 | | 1.98 |
|  | 3.79-243.1711 | 3.79 | 0.14 | 17337.03 | 3.32 | 3.79 | 0.14 | 17343.68 | 13.95 | 3.80 | 0.20 | 20167.31 | | 6.94 |
|  | 11.73-500.2772 | 11.73 | 0.05 | 429921.21 | 2.62 | 11.73 | 0.04 | 248440.33 | 13.93 | 11.74 | 0.05 | 306171.80 | | 1.87 |
|  | 12-526.2925 | 11.99 | 0.03 | 375701.41 | 2.28 | 11.99 | 0.04 | 207547.22 | 12.54 | 12.00 | 0.03 | 259152.30 | | 3.12 |
|  | 12.39-478.2925 | 12.39 | 0.04 | 318361.60 | 4.33 | 12.39 | 0.06 | 186217.20 | 12.49 | 12.40 | 0.06 | 224715.10 | | 4.86 |

**SUPPLEMENTARY TABLE 3**Potential biomarkers of inflammation in (R)-salbutamol-treated LPS-induced macrophages obtained from UHPLC/ESI-TIMSTOF-MS/MS

| **NO** | **RT (min)^a^** | **m/z** | **Mode** | **Biomarker identification** | **Formula** | **Trend ^b^** | **Trend ^c^** | **Trend ^d^** | **Related pathway** |
| --- | --- | --- | --- | --- | --- | --- | --- | --- | --- |
| 1 | 8.87 | 473.0449 | ^e^ P+N | Deoxyribose-5-phosphate | C_5_H_11_O_7_P | ↑** | ↓** | - | Pentose phosphate pathway |
| 2 | 10.73 | 468.3084 | P | LysoPC(14:0) | C_27_H_48_NO_7_P | ↑** | ↓** | - |  |
| 3 | 11.38 | 494.3239 | P | LysoPC(16:1(9Z)) | C_24_H48NO_7_P | ↑** | ↓** | - | Glycerophospholipid metabolism |
| 4 | 11.72 | 526.2929 | P | LysoPE(0:0/22:6(4Z,7Z,10Z,13Z,16Z,19Z)) | C_27_H_44_NO_7_P | ↑** | ↓** | - |  |
| 5 | 11.73 | 502.2927 | P | LysoPE(0:0/20:4(8Z,11Z,14Z,17Z)) | C_25_H_44_NO_7_P | ↑** | ↓** | - |  |
| 6 | 12.00 | 528.3077 | P | LysoPE(0:0/22:5(7Z,10Z,13Z,16Z,19Z)) | C_25_H_48_NO_7_P | ↑** | ↓* | - |  |
| 7 | 12.10 | 496.3387 | P | LysoPC(16:0) | C_24_H_50_NO_7_P | ↑** | ↓** | - |  |
| 8 | 12.34 | 496.3396 | P | LysoPC(16:0) | C_24_H_50_NO_7_P | ↑** | ↓** | - |  |
| 9 | 12.65 | 480.3085 | P | LysoPE(18:1(11Z)/0:0) | C_23_H_46_NO_7_P | ↑** | ↓** | - | Glycerophospholipid metabolism |
| 10 | 18.14 | 122.0966 | P+N | Phenylethylamine | C_8_H_11_N | ↑** | ↓** | - | Phenylalanine metabolism |
| 11 | 18.38 | 678.5066 | P | PE(15:0/16:0) | C_24_H_50_NO_7_P | ↑** | ↓** | - |  |

^a^ RT: retention time of the components; ^b^↑ represents the upregulation of the metabolites in the model group in comparison to the control group; ^c^↓ represents the downregulation of the metabolites in the (R)-salbutamol group comparison to the model group; ^d^- represents no significant change in the metabolites of the model group in contrast to those of the ICI-118551 group. ^e^ P and N represent ESI (+) and ESI (-). ** indicates a significant change (p < 0.01), * indicates a significant change (p < 0.05).
